# Fabrication, Structural Characterization, and Photon Attenuation Efficiency Investigation of Polymer-Based Composites

**DOI:** 10.3390/polym16091212

**Published:** 2024-04-26

**Authors:** Sitah F. Alanazi, Norah M. Alotaibi, Mohammed Alsuhybani, Nassar Alnassar, Fahad I. Almasoud, Mansour Almurayshid

**Affiliations:** 1 Department of Physics, College of Science, Imam Mohammad Ibn Saud Islamic University (IMSIU), Riyadh 11642, Saudi Arabia; sfenazi@imamu.edu.sa (S.F.A.); nomohalotaibi@sm.imamu.edu.sa (N.M.A.); nalnassar@imamu.edu.sa (N.A.); 2 Nuclear Technologies Institute, King Abdulaziz City for Science and Technology (KACST), Riyadh 11442, Saudi Arabia; sohybani@kacst.gov.sa (M.A.); fmasaud@gmail.com (F.I.A.); 3 Department of Soil Sciences, College of Food and Agricultural Sciences, King Saud University, Riyadh 12372, Saudi Arabia

**Keywords:** polymers, HDPE, X-ray shielding, silicon nanoparticles, radiation protection

## Abstract

Experiments have assessed various polymer composites for radiation shielding in diverse applications. These composites are lighter and non-toxic when compared to lead (Pb), making them particularly effective in diagnostic imaging for shielding against low-energy photons. This study demonstrates the fabrication of four composites by combining a base material, specifically a high-density polyethylene (HDPE) polymer, with 10% and 20% silicon (Si) and silicon carbide (SiC), respectively. Additionally, 5% molybdenum (Mo) was incorporated into the composites as a heavy metal element. The composites obtained were fabricated into 20 disks with a uniform thickness of 2 mm each. Discs were exposed to radiation from a low-energy X-ray source (32.5–64.5 keV). The chemical and physical properties of composites were assessed. The shielding ability of samples was evaluated by determining the linear and mass attenuation coefficients (μ and μ_m_), radiation protection efficiency (RPE), half-value layer (HVL), and mean free path (MFP). According to our findings, supplementing HDPE with additives improved the attenuation of beams. The μ_m_ values showed that composite X-ray shielding characteristics were enhanced with filler concentration for both Si and SiC. Polymer composites with micro-molecule fillers shelter X-rays better than polymers, especially at low energy. The HVL and MFB values of the filler are lower than those of the pure HDPE sample, indicating that less thickness is needed to shield at the appropriate energy. HC-20 blocked 92% of the incident beam at 32.5 keV. This study found that increasing the composite sample thickness or polymer filler percentage could shield against low-energy radiation.

## 1. Introduction

The increasing use of ionizing radiation across various scientific fields has heightened the risk of severe damage and health complications, such as radiation poisoning and burns. Consequently, there is a pressing need for the precise control and regulation of radiation [1]. Exploring radiation shielding for X-rays, gamma rays, and neutrons stands as a pivotal focus within the realm of radiation physics. Essential parameters like the mass attenuation coefficient and its derivative play a fundamental role in choosing materials for shielding against X-ray and gamma radiation [2,3].

Developing a radiation shielding system with optimal effectiveness in medical applications stands as a pivotal objective, emphasizing non-toxic, eco-friendly, and lightweight materials. Lead, often chosen for its high atomic number, density, and effective attenuation coefficients, interacts with high-energy photons like X-rays and gamma rays, leading to their absorption and scattering by atomic nuclei and electrons, resulting in reduced energy and intensity [4,5]. Yet, Pb drawbacks, notably its toxicity and weight, call for substituting it with non-toxic shielding materials [6]. Concrete is often utilized as an alternative to metals for shielding against beams in the diagnostic energy range due to its widespread availability and affordability [7]. However, this requires the construction of large-volume shields. Hence, scientists are exploring alternatives that not only offer excellent radiation reduction, but also align with the environmental sustainability, ease of molding, and cost-effectiveness criteria met by polymers. Numerous substitute substances, such as tungsten, boron, gadolinium, and bismuth, alongside a variety of polymer composites incorporating these elements, have been identified for this purpose [8,9,10,11,12,13,14].

Bismuth, classified as a heavy metal, offers shielding properties akin to lead, but with lower toxicity levels. Studies have delved into the potential of bismuth compounds like bismuth oxide, bismuth carbonate, and bismuth sub-nitrate for X-ray shielding applications [13,15]. Tungsten, a heavy metal capable of effectively absorbing photon radiation, and boron, proficient in neutron absorption, are also crucial elements to consider, especially in shielding against neutron radiation [16,17,18]. Additionally, research has focused on employing polymer composites for X-ray attenuation, expanding beyond the aforementioned materials. Interest in using polymers as matrices and reinforcing them with fillers has sparked significant attention in radiation protection research [5,19,20]. Efforts to develop innovative polymer composites as efficient shields against ionizing radiation have intensified. These composites typically rely on materials with high atomic numbers or elements other than lead, such as cerium oxide, tungsten oxide, and erbium phosphate, to offer X-ray shielding [21,22]. 

Polymers doped with heavy minerals or lead-free metals like tungsten, coupled with their oxides or salts, possess favorable traits for radiation shielding, including a low weight, high oxidation resistance, and plasticity. High-density polyethylene (HDPE), a prevalent polymer with a density surpassing 0.94 g/cm^3^, has found extensive use in radiation shielding due to its excellent protective properties and straightforward manufacturability. Notable for its chemical resistance and low coefficient of friction, HDPE facilitates easy machining and shaping into intricate configurations [23,24,25]. In a recent study, a radiation shield composed of polymers was examined. The study proposed seven potential shielding materials, comprising ethylene vinyl acetate (EVA) copolymers embedded with silicon, silicon carbide, and boron carbide [26]. The efficacy of these composite materials in shielding was evaluated via subjecting them to photon beams and analyzing parameters such as µ, µ_m_, HVL, MFP, and RPE. The measured µ_m_ values were compared to calculated ones, and the analysis of the experimental RPE findings demonstrated that polymer composites containing Si (30%) effectively blocked 90–91% of X-rays at around 80 kV, at a sample thickness of 12 cm.

In this work, we proposed Pb-free fillers embedded in a polymer to be used as a shielding material against low-energy X-ray radiation. Four different HDPE-based composites were prepared with varying weight percentages of Si and SiC as follows: 10% and 20% and fixed Mo 5%. The characterization process encompasses an extensive evaluation of both chemical and physical properties, in addition to assessing the shielding capabilities of these materials. This assessment includes determining crucial parameters, such as the µ and µ_m_, RPE, HVL, and MFP, for the prepared composites. Such a comprehensive analysis aims to thoroughly understand and quantify the shielding effectiveness of these Pb-free composite materials against low-energy X-ray radiation.

## 2. Materials and Methods

The present study involved an assessment of various aspects related to HDPE + EVA polymers based composites, including its fabrication, density measurements, mechanical characterization through tensile testing, and the determination of its thermal behaviors, as well as µ and µ_m_. In this work, composites were fabricated based on a HDPE (SABIC, Riyadh, Saudi Arabia) and EVA, (DUPONT, Delaware, DE, USA). The composites were embedded by different weight ratios of a Pb-free materials, such as Mo powder, less than 5 micron purity 95% (Goodfellow, Pittsburgh, PA, USA), Si as 150 micron with a purity of about 97% (Goodfellow, Pittsburgh, PA, USA), and SiC as 75 micron purity 98% (Goodfellow, Pittsburgh, PA, USA). We chose Mo, along with Si and SiC, for our polymer composites due to their effective radiation attenuation, mechanical synergy, cost efficiency, and lower toxicity, particularly as Mo is well suited for shielding within the low-energy range used in this study. We chose just a 5% Mo concentration for our composite to create an affordable shield comparable to the conventional concrete used in hospitals, optimizing the balance between enhanced radiation absorption and maintaining the polymer’s physical properties. The addition of EVA to HDPE aims to enhance several critical properties of the resulting composite, including increased flexibility, improved thermal resistivity, heightened resistance to environmental stress cracking, enhanced electrical resistance, and the better dispersion of filler particles within the matrix [27,28]. The preparation of our samples will be explained in the following section.

### 2.1. Samples Preparation 

Materials were mixed at a specific ratio, and the composite samples were provided with the codes as shown (Table 1). The preparation process was initiated by heating the HDPE polymer in the chamber of a Brabender Plasticorder mixer (Brabender, Duisburg, Germany) to a temperature range between 190 °C for a minute. The EVA polymer was then added for another minute, allowing the polymer to melt. Finally, Mo and Si or SiC were added slowly in different ratios to enhance the homogeneity of the compound. During the mixing process, the speed of the screw mixer is constant, at 40 rpm, with a temperature of 190 °C for 10 min. The compound was then transferred to the two roll mill (Brabender, Duisburg, Germany), which was pre-set at 190 °C and 20 rpm for 4 min in order to form a homogeneous mixture that is uniformly distributed within the polymer matrix used to acquire a plain sheet. Then, 44.5 g, 47.9 g, 45.4 g, and 49.2 g were placed in the frame of stainless steel that consists of two double layers of a 2 mm stainless steel and a PET film to protect the composite. The frame containing the compound is then placed in a hot press machine (Collin, Maitenbeth, Germany). With a gradual rise in pressure and temperature needed to obtain a flat sheet with free bubbles, the samples were then removed and cooled to room temperature. Finally, discs with a diameter of 2.5 cm and a dog bone shape were cut using a Ceast cutting machine (Instron, Pianezza, Italy) in order to evaluate a mechanical and shielding test. The thickness of the disc was measured using a caliper (Mitutoyo, Aurora, IL, USA).

Table 1 displays the fabricated polymer composite, and the composite samples were provided, named Pure HDPE, which contained only 85% HDPE + 15% EVA polymers without any filler. All the prepared composite materials were based on 15% EVA, plus either 60% or 70% HDPE. The remaining weight percentages were comprised of fillers as follows: 10% Si and 5% Mo in sample HS-10, 20% Si and 5% Mo in sample HS-20, 10% SiC and 5% Mo in sample HC-10, 20% SiC and 5% Mo in sample HC-20, and 5% Mo in sample H-Mo.

### 2.2. Density Measurement

The density measurement in this work was performed with an analytical balance (Mettler Toledo GmbH, Greifensee, Switzerland), based on the Archimedes’ principle. The weight of the samples was measured in both air and ethanol across five trials per sample, and the average value was calculated. Then, calculate the density according to the following equation (Equation (1)).

(1) $$\rho =\frac{m˳}{m˳-m_{l}}\left(\;\rho_{l}-\rho ˳\right)+\rho ˳$$

where ρ is the density of the sample, m˳ is the mass of the sample in air, $m_{l}$ is the mass of the sample in the auxiliary liquid, $\rho_{l}$ is the density of the auxiliary liquid, and ρ˳ is the density of air. The temperature of the liquid should be taken into account when determining the density with an accuracy of more than 1%. Here, ethanol was used and had a temperature between 16 °C to 18 °C, and each of them had a different density.

### 2.3. The Mechanical Characterization of Fabricated HDPE Composite Samples 

#### Tensile Testing

The most common testing machines are comprehensive testers used for tensile, compressive, or bending tensions. Tensile testing involves fitting the sample into a tensile machine and subjecting it to controlled tension until it breaks. The tensile test can be used to determine the maximum tensile strength, fracture strength, maximum stretching, and area reduction. The test was performed using a Universal Testing System 5982 (Instron, Norwood, MA, USA) with a capability of 100 kN; with this device, all mechanical properties resulting from tension or compression can be measured at normal and high temperatures, as it contains an oven for heating and cooling up to 350 degree Celsius, which can be installed during the measurements. The device displays all changes of tensile strength and elongation, and performs various analyses by means of the program connected with the device. The test performs according to ASTM D638 [29]. Using dumbbell samples cut from pressed sheets of 2 mm thickness, the sample has enlarged ends or shoulders for gripping, and the attachment areas at each end of the specimen should also be aligned with the axis of the tape, at a crosshead speed of 50 mm min^−1^. The sample is usually made in multiple samples for testing; our results are an average of at least three to four measurements. Here, the tensile strength was calculated when the compound was broken.

### 2.4. Thermal Analysis by TGA and Combustion Testing

The thermal behavior of the sample was evaluated using a thermogravimetric analyzer (Perkin Elmer, Shelton, CT, USA). TGA was employed to study the thermal behavior because it reflects the weight loss of the composites with the temperature. Each sample was heated from room temperature to 700 °C at a rate of 10 °C min^−1^, under nitrogen gas conditions. The combustion test is proposed to confirm the homogeneity of the additives within the polymer by burning the samples, and using the remaining samples as a weight to compare to the theoretical weight. The specimens for this burning test were cut to three measurements with a weight of 5 to 6 g for each measurement of each sample at 500 °C for 1 h.

### 2.5. Linear and Mass Attenuation Coefficients Determination

The Lambert-Beer law provides an equation that establishes a relationship between the μ_m_, residual radiation intensity (*I*), initial radiation intensity (*I*_0_), and the thickness of the material (x) [16,30]. The equation describes the relationship between the residual radiation intensity (*I*) and the initial radiation intensity (*I*_0_) after passing through a thickness (x) of a material. The term *μ* represents the linear attenuation coefficient of the material [31].

(2) $$\frac{I}{I_{0}}={exp}^{\left(-\mu_{\;}x\right)}$$

The expression of the *μ_m_* can be represented in relation to the *μ* and the ρ of the material. The *µ* is defined as the proportion of radiation intensity that is lost per unit thickness of the material.

(3) $$\mu_{m}=\frac{\mu }{\rho }$$

The material-specific property known as the *μ_m_* is contingent upon both the atomic composition of the material and the energy of the incident radiation. In general, materials that possess a greater atomic number and density tend to demonstrate greater *μ_m_* coefficients when subjected to a specific energy of radiation. The application of the Lambert-Beer law for the purpose of ascertaining the *μ_m_* has been expounded upon in numerous scholarly publications, as exemplified by the aforementioned pair of investigations [30].

The estimated RPE of all HDPE compound materials were determined across a range of energies, specifically from 32.5 to 64.5 keV. This estimation was calculated through the use of Equation (4) [32,33].

(4) $$RPE=\left(1-e^{-\mu }\right)\times 100\left(\%\right)$$

The HVL of various HDPE composite materials was determined using Equation (5) for different energy levels ranging from 32.5 to 64.5 keV.

(5) $$\mathrm{HVL}=\frac{Ln(2)}{µ}$$

The MFP measures the average photon distance between two subsequent encounters. It can be shown from Equation (6) that this parameter is inversely proportional to the *µ*.

(6) $$MFP=\frac{1}{\mu }$$

#### 2.5.1. Experimental Setup

**Figure 1:**
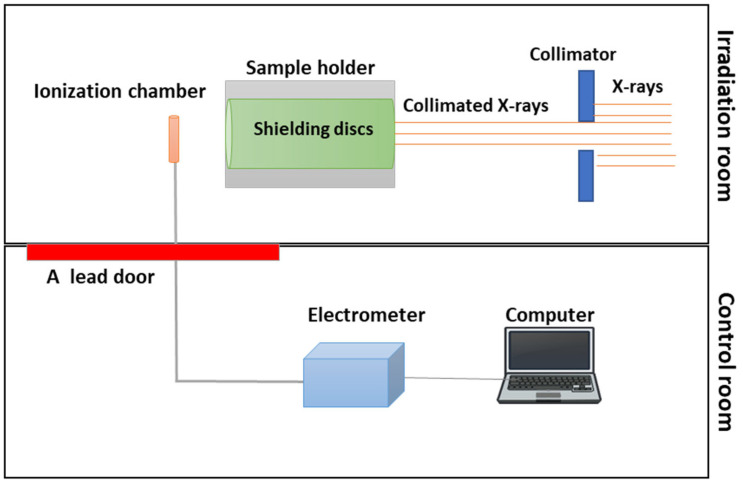
Sketch of the experimental setup showing the X-ray beam penetrate the sample, as detected by the ionization chamber detector system.

The experiment was performed at the Radiation Calibration Laboratory in King Abdulaziz City for Science and Technology (KACST), Saudi Arabia. The main goal was to determine the effectiveness of samples manufactured for this study in shielding and protecting against radiation. An X-ray source was used (32.5–64.5 keV) to deliver radiation beams through a collimator to the sample placed in the holder, 100 cm away from the radiation source (Figure 1).

We controlled the process remotely through the control room, which is separated by a thick lead door around the irradiation bunker. A sample holder made of pb was placed between the radiation source and the detector, allowing us to measure the radiation beams sent through the disks via the Exradin A3 spherical ionization chamber detector (Standard Imaging, Middleton, WI, USA). An electrometer (PTW, Freiburg, Germany) in the control room was also linked to an ionization chamber detector in order to process the signal. The ionization chamber and the electrometer were controlled with a computer using the PTW UNIDOS software package (Version 1.3).

#### 2.5.2. X-ray Attenuation Characteristics of Composite Materials 

The attenuation coefficient is a basic quantity used in the calculation of the penetration of materials by energy beams, and it is of great importance in radiation shielding. We studied the µ_m_ of composite polymer samples, as well as the effect of each added Si and SiC on the polymer at different concentrations, through the use of X-rays at low energies from 32.5 keV to 64.5 keV. The radiation travels through the center of the detector 50 cm from the radioactive source, and the exposure time is set to 10 s. The irradiation process started with the assumption that (*I*_0_) is the intensity of the incident radiation measured without a disc. Then, 13 discs were positioned in the sample holder in the central beam axis in order to measure the transmitted intensity (*I*). By considering (*I*_0_) and (*I*), one can calculate the relative dose (%), given that the slope represents the value of the µ. Accordingly, the µ_m_ was determined by dividing the attenuation coefficient by the sample density. The same previous procedure was repeated with the change in the X-ray energy on different samples, as shown in Table 2.

In order to assess the efficacy of the shielding, the following aspects were examined: The efficiency of the RPE serves as an accurate indicator of the shielding ability for composite samples. This is achieved via determining the measured intensity both with and without the sample, as well as considering the HVL and MFP, gaining a deeper understanding of the attenuation ability. It is important to note that the HVL and MFP are significantly influenced by the energy involved, and lower values indicate a more effective shielding capability.

## 3. Results and Discussion

### 3.1. Density Measurements

Table 3 presents the theoretical and experimental density values for varying concentrations of Si and SiC, along with the corresponding error rates. It is widely acknowledged that the overall density of a composite material filled with particles relates directly to the density of its individual constituent particles. Table 3 demonstrates that the overall density of the composite material exhibited an increase with the increase in the loading of Si or SiC. The observed increment in density can be attributed to the varying densities of Si and SiC, which are notably greater than those of HDPE. The current experimental investigation included a density range of 1.07–1.15 g cm^−3^ for HDPE composites. The maximum density recorded was 1.15 g cm^−3^ for HDPE containing 20 wt.% of SiC, leading to a notable 23% rise in density when compared to pure HDPE with a density of 0.95 g cm^−3^. Conversely, the addition of 10 weight percent of Si and SiC into HDPE composites resulted in a reduced density of approximately 1.07 g cm^−3^. This value was lower than the estimated densities of 1.03 g cm^−3^ and 1.05 g cm^−3^ for Si and SiC, respectively. Furthermore, Table 3 indicates that there was no significant difference between the experimental and calculated values. Furthermore, one of the measurements that verifies the homogeneity of the additives throughout the polymer is regarded as an essential property that impacts other properties such as shielding, among others.

### 3.2. The Mechanical Characterization

**Figure 2:**
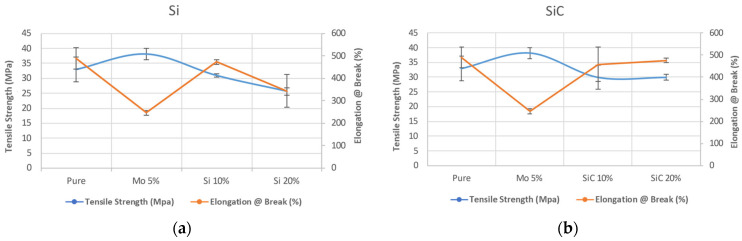
The mechanical properties of HDPE when (**a**) the samples were filled with Si or when (**b**) the samples were filled with SiC.

The mechanical properties of HDPE composites, namely tensile strength and the elongation at the break, exhibit variations in response to the concentration of Si and SiC, as illustrated in Table 4. The pure HDPE exhibited a tensile strength of 32.95 MPa. However, the addition of fillers at varying loading resulted in a decrease in tensile strength. Specifically, the tensile strength values were 30.93 MPa and 25.62 MPa for HS-10 and HS-20, respectively. Similarly, the addition of the SiC fillers resulted in tensile strength values of 30.02 MPa and 29.99 MPa for HC-10 and HC-20, respectively. The mechanical properties of various composites were observed to decrease with the increasing loading of Si and SiC fillers. The tensile strength and elongation at the break of the composite material exhibited a decrease in the presence of silicon as the weak zone, as already defined, increased in size with a corresponding increase in the filler content. Similar observations were made in the case of tensile behavior upon the addition of SiC into HDPE. The findings indicate a reduction in elongation for HC-10 and HC-20 in comparison to the pure sample, with respective values of 473 and 344 Pa. However, for SiC, the elongation value of HC-10 exhibited a decrease relative to HC-20, with respective values of 458 and 476. Conversely, the addition of only Mo without any additional filler resulted in a significant increase in the tensile strength of the material in comparison to pure HDPE. Specifically, the tensile strength was measured at 38.16, while the elongation at the break for H-Mo was observed to decrease to 246, see Figure 2.

### 3.3. Thermal Stability

**Figure 3:**
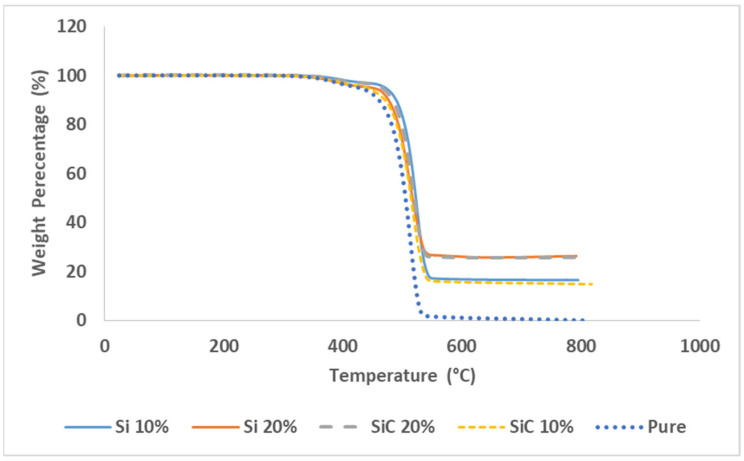
TGA results of the fabricated samples.

This study represents a comprehensive assessment of the thermal properties within the temperature range from 25 to 800 degree Celsius. Figure 3 displays the TGA curves of HPDE in its pure form and composites that contain Si or SiC in different weight percentages, up to a maximum of 20 wt.%. According to the TGA curve illustrated in Figure 3, it can be concluded that the stability of pure HDPE remains unaltered up to a temperature of 451.20 °C, without any significant loss of mass. At a temperature of 550 °C, HDPE undergoes complete thermal decomposition. The addition of inorganic fillers results in a significant enhancement of the thermal stability of the corresponding polymers. The findings of the present study demonstrate that an increase in the Si concentration leads to a reduction in the rate of the mass loss observed in the composites. The thermal degradation of the composites started within the temperature range from 360 to 465 °C, resulting in mass loss. Table 5 presents information regarding the thermal stability of different composites. Based on the information presented in Table 5 and Figure 3, it can be concluded that the thermal stability of HDPE is generally lower when compared to that of HDPE + Si or HDPE + SiC composites. This observation provides clear evidence that the thermal stability of diverse inorganic compounds is greater than that of pure polymers. Moreover, the improved thermal stability of said composites may be linked to an increase in the Si and SiC density. The experimental results indicate that the addition of Si (10%, 20%) and SiC (10%, 20%) into the HDPE composite resulted in a significant increase in the char yield. Specifically, the char yields of the HDPE + Si (10%, 20%) and the HDPE + SiC (10%, 20%) composites were found to be 16.81%, 27.29%, 15.75%, and 25.61%, respectively, higher than that of pure HDPE at 600 °C, which showed a char yield of 1.15%. The values reported indicate that the distribution of Si and SiC within the polymer was significant, resulting in a proximity to the initial weight percentage applied. This was determined through a combustion test, as demonstrated in Table 6, which displays the combustion characteristics of HDPE, including various loads of Si or SiC.

### 3.4. Linear and Mass Attenuation Coefficient Measurement

**Figure 4:**
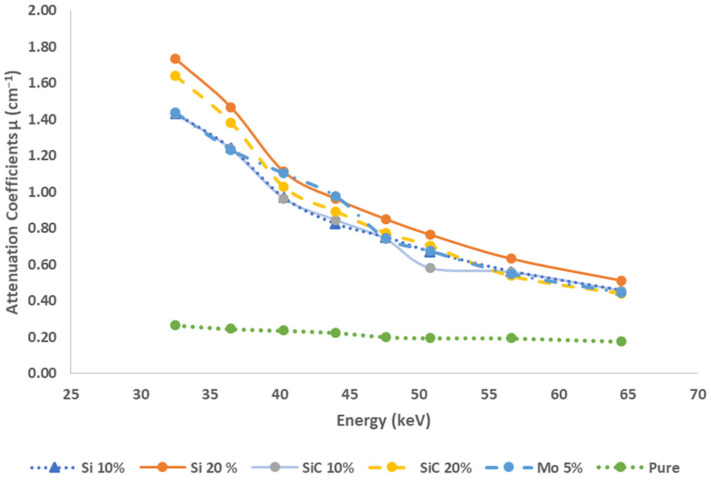
The measured linear attenuation coefficient against the incident beam energy.

Figure 4 illustrates the attenuation coefficients of the prepared samples, utilizing polymer HDPE when subjected to a low energy X-ray beam ranging from 32.5 keV to 64.5 keV. The objective of this experiment was to determine the values of *I* and *I*_0_, while maintaining the samples in the carrier. This information is important for calculating the corresponding µ at various energy levels. Based on the data presented in the figure, it can be observed that the μ exhibits a decreasing pattern as the energy increases. Furthermore, the µ exhibits an increase when integrating silicon and silicon carbide particles. Specifically, for HS-10 and HS-20, the coefficients were measured to be 1.34 ± 7 and 1.52 ± 0.59, respectively. Similarly, for HC-10 and HC-20, the coefficients were found to be 1.34 ± 5.25 and 1.43 ± 3.43, respectively. These measurements were conducted at an energy level of 32.5 keV for each of these materials. In our investigation, including various energy levels of incident beams, we have observed that the HDPE composite, characterized by a silicon ratio of 20%, exhibits greater attenuation when compared to other composites with lower ratios. Furthermore, it demonstrates higher attenuation than the compound with a silicon carbide ratio of 20%, with a measured value of 1.52 ± 0.59 for HS-20. It ensures that the addition of silicon filler content leads to a corresponding increase in attenuation when compared to the pure polymer. Hence, the efficiency demonstrates an improvement when compared to the pure polymer, specifically within the lower energy spectrum (32.5 keV–44 keV). This enhancement can be attributed to the prevailing influence of photoelectric absorption, which is dependent upon both the energy level of photons and the atomic number of materials. In the specified energy range from 47.6 keV to 64.5 keV, it is observed that the µ decreases as the energy increases for all composites, which is probably because the Compton attenuation is the dominant mechanism. In order to validate the findings obtained in the laboratory, a comparison was made between these results and the results of the composite samples obtained from the XCOM database provided by the National Institute of Standards (NIST) [34], as presented in Table 7. For instance, for the SiC 20% composite, the measured attenuation at an energy level of 50.8 keV was determined to be 0.61, while the calculated attenuation at the same energy level was found to be 0.56.

$$Percentage\;error=\frac{\left|0.61-0.56\right|}{0.56}\times 100\%=8.9\%$$

The results indicate that the measured and calculated values are approximately equivalent, with a minor percentage error due to the experimental procedures. At lower energy levels, such as 32.5 keV, there is a significant difference in the attenuation values. This difference highlights the considerable significance of silicon and silicon carbide as filler materials for HDPE polymers in the present study. These materials have demonstrated their efficacy in radiation protection at lower energy levels, which are extensively employed in research centers, as well as in hospitals, for diagnostic and therapeutic purposes.

### 3.5. Radiation Protection Efficiency (RPE)

Table 8 illustrates that the sample containing 20% SiC exhibits the highest RPE values, specifically 92.31, 89.20, 86.25, 82.92, 78.91, 77.57, 68.61, and 61.29, whereas the pure HDPE sample demonstrates the lowest RPE values, which are 40.77, 38.46, 37.47, 36.13, 32.93, 32.10, 32.28, and 30.14, respectively, across a range of energies, spanning from 32.5 to 64.5 keV. The data obtained also revealed a decline in the RPE with increasing photon energy. It is evident that, at higher energies, there is a slight decrease in the RPE. This observation suggests that the efficiency ratio of radiation protection is greater for low photon energies when compared to high energies. This aligns with the main objective of our current study. The findings obtained from the study indicate that the composite materials examined exhibited a maximum efficiency of 92.31% (RPE) when the shielding materials contained 20% SiC at an energy level of 32.5 keV. Conversely, the lowest efficiency of approximately 30.14% was observed in the pure HDPE sample at a photon energy of 64.5 keV. The RPE is not solely influenced by the higher concentration of additive materials within the polymer matrix; sample thickness also plays a pivotal role in determining the overall attenuation characteristic. Variations in the sample thickness directly influence the ability of the composite materials to attenuate the X-rays. Thicker samples tend to offer increased attenuation due to the higher number of atoms or molecules encountered by the X-rays, resulting in greater absorption or scattering.

### 3.6. Half-Value Layer (HVL)

**Figure 5:**
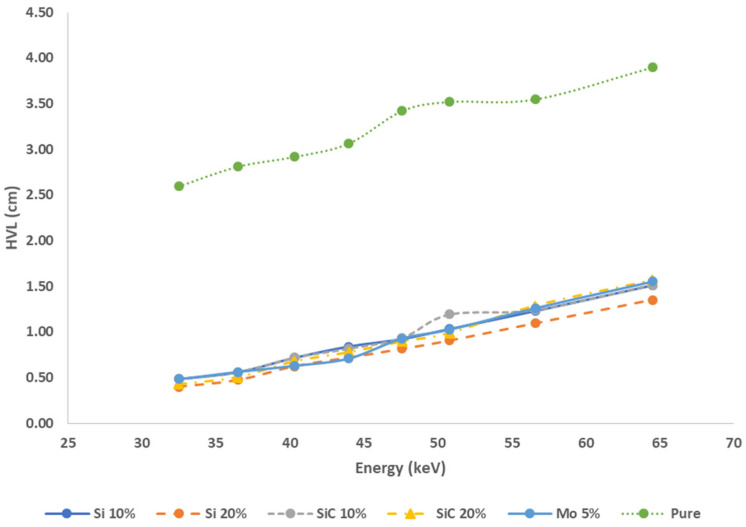
HVL values against the energy for the composite samples.

The results indicate that, as the concentration of Si or SiC in the samples increases, the HVL decreases. Specifically, for HS-10 and HS-20, the HVL values were found to be 0.48 and 0.40, respectively. Similarly, for HC-10 and HC-20 with SiC, the HVL values were 0.48 and 0.42, respectively. Additionally, for the H-Mo samples, the HVL was consistently measured at 0.48 cm for an energy level of 32.5 keV. The dose rate is reduced by half of its initial value as a result of the decrease in the HVL. Consequently, it can be observed that the sample with a Si content of 20% exhibits the lowest HVL, while the pure HDPE sample demonstrates the highest HVL, measured at different energy levels ranging from 32.5 to 64.5 keV, yielding respective values of 2.60, 2.81, 2.91, 3.06, 3.42, 3.52, 3.55, and 3.89 cm. The data obtained from the experiment also demonstrated a positive correlation between the HVL and photon energy. This relationship is illustrated in Figure 5, where it can be observed that the HVL shows a gradual increase at higher energy levels.

### 3.7. Mean Free Path (MFP)

**Figure 6:**
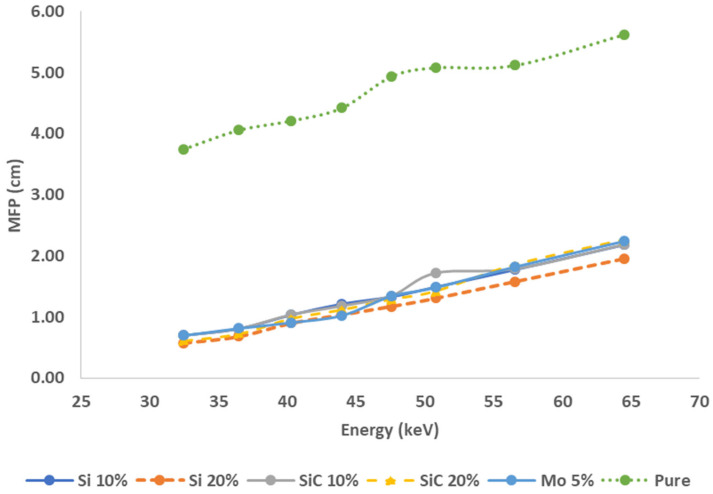
Variation of the MFP with energy for the HDPE composite sample.

The MFP results might validate the effectiveness of the investigated HDPE compound for X-ray shielding applications. It was discovered that the MFP tends to increase fast with increased energy. Figure 6 clearly demonstrates that the MFP decreases as the photon energy decreases, and increases as the photon energy increases. It can be seen that the excess Si and SiC content in the polymer composite has a significant effect on the shielding efficiency of this HDPE polymer. This difference may thus be expressed in terms of photon interactions in the given energy range for the composite samples. The MFP values are lower the denser the sample is, and the photoelectric absorption had a considerable effect on the radiation protection parameter at low energies, but the Compton process predominated at high energies. Consequently, pure HDPE composites have the greatest MFPs, which are 3.74, 4.04, 4.21, 4.42, 4.93, 5.08, 5.12, and 5.62 cm, respectively. Whereas, the samples containing 20% Si composites have the lowest MFPs, which are 0.58, 0.68, 0.90, 1.04, 1.17,1.31, 1.58, and 1.95 cm, respectively, at various energies, ranging from 32.5 to 64.5 keV for all of them, resulting in the highest densities and masses and the highest X-ray shielding capabilities among all of our suggested composites.

We have expanded our comparative analysis to include data concerning the HVL and MFP of various shielding materials at comparable energy levels. Table 9 illustrates that HDPE composites, enhanced with 20% silicon and 5% molybdenum, exhibit superior attenuation properties, demonstrating lower HVL and MFP values than the competing materials, particularly the commonly used concrete in room shielding for diagnostic energy range beams. Utilizing the data from the table, a comparison reveals that, at approximately 48 keV, a 6 cm-thick layer of our HDPE (75%) + Si (20%) + Mo (5%) composite would attenuate 99.41% of X-rays, significantly outperforming concrete, which attenuates only 76.58% under the same conditions, as calculated using Equation (2).

Experimental results show that a 4 cm-thick layer of this composite can attenuate 85–92% of X-rays at 32.5 keV. Improvements in the shielding effectiveness can be achieved by either increasing the shield thickness or boosting the filler content within the composites, making it an ideal option for shielding in diagnostic radiology or research facilities utilizing low-energy applications. This method decreases the reliance on costly and hazardous lead shields, and provides safer alternatives for protecting patients in low-energy radiation environments and for isolating sensitive detectors and electronic instruments.

## 4. Conclusions

This study developed lightweight and environmentally friendly polymer composites as effective X-ray shields, comparing them to heavier lead-based alternatives. We prepared four samples via mechanically mixing HDPE with 10% and 20% of Si and SiC, plus a constant 5% Mo, observing variations in density that correlate with the differing filler concentrations. The HDPE + SiC (20%) composite showed the highest density increase, approximately 20% greater than with pure HDPE. Our evaluation of attenuation properties revealed that the RPE decreases with a higher photon energy, yet improves with increased filler concentrations. Notably, composites with 20% Si and SiC demonstrated superior X-ray attenuation, especially the 20% Si composite, which showed the most effective shielding. The study confirmed that increasing the filler content enhances X-ray shielding, suggesting that adjusting the composite thickness or filler proportion could further optimize protection, particularly against low-energy radiation. Future research might explore these materials’ effectiveness at higher energy levels and their capabilities to reduce neutron radiation impacts.

## Figures and Tables

**Table 1:** The ratios of the materials used in the preparation of samples.

Sample	HDPE (%)	EVA (%)	Mo (%)	Si (%)	SiC (%)
Pure HDPE	85	15	0	0	0
HS-10	70	15	5	10	0
HS-20	60	15	5	20	0
HC-10	70	15	5	0	10
HC-20	60	15	5	0	20
H-Mo	80	15	5	0	0

**Table 2:** X-ray beam qualities used in this study.

Quality	Tube Voltage (kV)	Effective Energy (keV)	Total Filtration Al (mm)
RQR 3	50	32.5	2.46
RQR 4	60	36.5	2.68
RQR 5	70	40.3	2.83
RQR 6	80	44	2.99
RQR 7	90	47.6	3.18
RQR 8	100	50.8	3.36
RQR 9	120	56.6	3.73
RQR 10	150	64.5	4.38

**Table 3:** The density measurements for the sample prepared in this study.

Sample Code	Density (Experimental g cm^−3^)	Density (g cm^−3^)	Error %
Pure HDPE	0.95	0.92	3.50
HS-10	1.07	1.03	3.43
HS-20	1.14	1.11	2.86
HC-10	1.07	1.05	2.27
HC-20	1.15	1.14	0.61
H-Mo	1	0.97	3.23

**Table 4:** Results of the mechanical properties of the samples.

Sample Code	Tensile Strength (MPa)	Elongation @ Break (%)
Pure HDPE	33.0 ± 4.2	489 ± 48
HS-10	30.9 ± 0.5	473 ± 10
HS-20	25.6 ± 1.3	344 ± 72
HC-10	30.0 ± 4.0	458 ± 78
HC-20	30.0 ± 1.0	476 ± 10
H-Mo	38.12 ± 1.8	246 ± 11

**Table 5:** Thermal stability properties of HDPE with different ratios of fillers (Si and SiC).

Sample Code	Onset Temperature		The Remaining Weight at 600 °C %
Pure HDPE	451.20		1.15
HS-10	399.2	480.4	16.81
HS-20	360.2	461.8	27.29
HC-10	371.4	465.3	15.75
HC-20	361.8	465.1	25.69

**Table 6:** Combustion test of HDPE with different ratios of fillers (Si and SiC).

Sample Code	The Theoretical Values	The Experimental Values	Error %
HS-10	0.55	0.87	0.59
HS-20	1.09	1.42	0.29
HC-10	0.54	0.82	0.53
HC-20	1.17	1.50	0.29

**Table 7:** Mass attenuation coefficients of the HDPE (µ_m_ (cm^2^ gm^−1^)) of the fabricated samples, measured experimentally and compared with theoretical values using X-COM.

Sample	Energy (keV)	32.5	36.5	40.3	44	47.6	50.8	56.6	64.5
Pure HDPE	Exp	0.23 ± 0.09	0.21 ± 0.01	0.21 ± 0.08	0.20 ± 0.09	0.18 ± 0.01	0.17 ± 0.01	0.17 ± 0.01	0.15 ± 0.02
	Cal	0.26	0.24	0.23	0.22	0.21	0.21	0.2	0.19
HS-10	Exp	1.34 ± 0.07	1.16 ± 0.04	0.91 ± 0.06	0.77 ± 0.04	0.70 ± 0.01	0.63 ± 0.01	0.53 ± 0.01	0.43 ± 0.01
	Cal	1.44	1.11	0.9	0.74	0.63	0.56	0.46	0.37
HS-20	Exp	1.52 ± 0.01	1.29 ± 0.01	0.98 ± 0.03	0.85 ± 0.08	0.75 ± 0.01	0.67 ± 0.01	0.56 ± 0.01	0.45 ± 0.01
	Cal	1.53	1.17	0.94	0.78	0.66	0.58	0.47	0.38
HC-10	Exp	1.34 ± 0.05	1.16 ± 0.06	0.90 ± 0.02	0.79 ± 0.08	0.69 ± 0.01	0.54 ± 0.01	0.52 ± 0.01	0.43 ± 0.02
	Cal	1.41	1.09	0.88	0.73	0.62	0.55	0.45	0.36
HC-20	Exp	1.43 ± 0.03	1.20 ± 0.06	0.89 ± 0.02	0.78 ± 0.03	0.67 ± 0.05	0.61 ± 0.09	0.47 ± 0.02	0.39 ± 0.04
	Cal	1.48	1.13	0.91	0.76	0.64	0.56	0.46	0.37
H-Mo	Exp	1.44 ± 0.06	1.23 ± 0.01	1.10 ± 0.02	0.98 ± 0.03	0.75 ± 0.02	0.68 ± 0.02	0.55 ± 0.02	0.45 ± 0.02
	Cal	1.35	1.04	0.85	0.71	0.6	0.53	0.44	0.36

**Table 8:** The radiation protection efficiency (RPE) in (%) of the fabricated HDPE composites.

Energy (keV)	Pure HDPE	HS-10	HS-20	HC-10	HC-20	H-Mo
32.5	40.77	88.71	91.32	87.99	92.31	85.74
36.5	38.46	85.29	88.08	84.69	89.20	81.87
40.3	37.47	82.86	84.97	81.67	86.25	78.72
44	36.13	79.12	81.42	78.02	82.92	75.05
47.6	32.93	75.76	77.77	73.99	78.91	71.38
50.8	32.10	72.17	74.38	68.13	77.57	68.08
56.6	32.28	65.68	68.17	64.52	68.61	60.88
64.5	30.14	58.08	59.92	56.89	61.29	53.51

**Table 9:** Comparison of the measured HVL and MFP results with other materials in the literature, using approximately 48 keV beams.

Study	Composites	HVL (cm)	MFP (cm)
This study	HDPE (75%) + Si (20%)+ Mo (5%)	0.81	1.29
Alshareef et al., 2023 [32]	HDPE (85%) + ZnO (15%)	1.19	1.71
Almurayshid et al., 2021 [26]	EVA(70%) + Si (30%)	2.35	3.39
(Bashter, 1997) [35]	Concrete (Portland cement)	2.86	4.137

## Data Availability

All data are contained within the article.
